# A Comprehensive Analysis of the Expression Profiles of KCTD Proteins in Acute Lymphoblastic Leukemia: Evidence of Selective Expression of KCTD1 in T-ALL

**DOI:** 10.3390/jcm12113669

**Published:** 2023-05-25

**Authors:** Lorena Buono, Concetta Iside, Giovanni Pecoraro, Antonia De Matteo, Giuliana Beneduce, Roberta Penta de Vera d’Aragona, Rosanna Parasole, Peppino Mirabelli, Luigi Vitagliano, Marco Salvatore, Giovanni Smaldone

**Affiliations:** 1 IRCCS SYNLAB SDN, Via E. Gianturco 113, 80143 Naples, Italy; 2 Department of Pediatric Hemato-Oncology, Santobono-Pausilipon Children’s Hospital, AORN, 80122 Naples, Italy; 3 Institute of Biostructures and Bioimaging, C.N.R., 80134 Napoli, Italy

**Keywords:** KCTD proteins, RNA-seq analysis, childhood acute lymphoblastic leukemia, biomarker, diagnostics

## Abstract

**Simple Summary:**

Acute lymphoblastic leukemia is the most frequent tumor in childhood age. Recently, we associated KCTD protein family members with childhood ALL with particular reference to B-ALL. In this scenario, our aim was to assess the expression landscape of all KCTD family members in pediatric B-cell and T-cell leukemias compared with mononuclear cells derived from cord blood samples from healthy subjects. Our data allowed us to identify the KCTD1 protein as a potential new biomarker of T-ALL, raising interest for future clinical investigations and validating its role not only as a diagnostic marker, but also as a new potential therapeutic target.

**Abstract:**

Acute leukemia is the most common pediatric cancer. In most cases, this disease results from the malignant transformation of either the B-cell (B-ALL) or, less frequently, T-cell progenitors (T-ALL). Recently, a marked overexpression of KCTD15, a member of the emerging class of the potassium (K) channel tetramerization domain-containing proteins (KCTDs) has been detected in both patients and continuous cell lines as in vitro model systems. Because there is growing evidence of the key, yet diversified, roles played by KCTDs in cancers, we here report an exhaustive analysis of their expression profiles in both B-ALL and T-ALL patients. Although for most KCTDs, no significant alterations were found in these pathological states, for some members of the family, significant up- and down-regulations were detected in comparison with the values found in healthy subjects in the transcriptome analysis. Among these, particularly relevant is the upregulation of the closely related KCTD1 and KCTD15 in T-ALL patients. Interestingly, KCTD1 is barely expressed in both unaffected controls and B-ALL patients. Therefore, not only does this analysis represent the first study in which the dysregulation of all KCTDs is simultaneously evaluated in specific pathological contexts, but it also provides a promising T-ALL biomarker that could be suitable for clinical applications.

## 1. Introduction

Acute leukemia is the most common type of cancer in childhood, but it affects all age groups [1]. Depending on the predominant lineage of the malignant cells, acute leukemia is generally distinct in lymphoblastic (ALL) and myeloid (AML) [2]. ALL accounts for nearly a quarter of all cases of pediatric cancer. Based on the type of cells that undergo malignant transformation, ALL cases are further classified as B-cell (B-ALL) or T-cell (T-ALL) [1]. B-ALL is the most common form of ALL, associated with distinct gene expression profiles and driven by three main types of initiating genetic alteration: (i) chromosomal aneuploidy, (ii) rearrangements that deregulate oncogenes or encode chimeric transcription factors, and (iii) point mutation [3]. On the other hand, T-ALL represents ~10–15% of newly diagnosed ALL cases, although this percentage significantly depends on age and/or ethnicity [4]. T-ALL is less frequent than B-ALL and has a worse prognosis, indeed about 20–30% of patients relapse, with a 5-year survival of approximately 20% [2,4]. Importantly, most of the clinical trials have historically shown that patients with T-ALL suffer worse outcomes than B-ALL patients [5,6]. Indeed, T-cell ALL blasts are generally more resistant to chemotherapeutic drugs than B-cell ALL blasts. Moreover, patients with B-cell ALL are more responsive to available targeted therapies [5]. Childhood T-ALL is featured by recurrent alterations mostly deregulating different pathways involving expression of T-lineage transcription factors, and cell-cycle control genes [7,8]. The etiopathogenic mechanisms leading to leukemic transformation are still largely unknown, but genetic, immunologic, viral, and environmental factors have been implicated [9,10,11]. Today, the classification of ALL into risk groups is based on lymphoblast morphology, immunologic markers, enzyme abnormalities, cytogenetic findings, and staining features in conjunction with clinical characteristics [12]. In this intricate scenario, we have very recently shown that KCTD15, a member of the potassium (K)-containing tetramerization domain (KCTD) protein family, is remarkably overexpressed in both B-ALL patients and cell model lines [13]. KCTD15-silencing experiments have demonstrated that this protein is likely involved in the upregulation of the NF-kB pathway, likely through the activation of the IKKβ kinase that specifically phosphorylates the NF-kB inhibitor IKBα, leading to its degradation [14]. Although KCTD proteins are traditionally considered as proteins involved in the physiopathology of the nervous systems, there is increasing evidence of their involvement in cancers [15]. Moreover, recent comprehensive predictive analyses of their three-dimensional structures have highlighted previously unreported similarities among the members of the family that can have functional implications [16]. Considering that NF-kB signaling is overactivated in many proliferative processes, including different types of leukemia [17], and the recently uncovered similarities among the members of the KCTD family, we investigated up- and down-regulations in both B-ALL and T-ALL patients of all members of the KCTD family. These analyses led to the identification of the remarkable over-expression of KCTD1 in T-ALL patients and in continuous immature T cell lines.

## 2. Materials and Methods

### 2.1. Study Population

The procedures followed in the present study have been approved by the local ethical committees of the IRCCS SYNLAB SDN and are in line with the Helsinki declaration [Ethical Committee IRCCS Pascale, Naples, Italy—protocol number 5/19 of the 19 June 2019, and the AORN Santobono-Pausilipon (Ethical Committee Cardarelli/Pausilion, Naples Italy—protocol number 07/20 of 3 June 2020)]. All participants provided informed assent through informed consent signed by both parents. Patients’ clinical data have been previously reported [18]. We used the St. Jude Children’s Research Hospital database for in silico analyses (https://platform.stjude.cloud/data/cohorts/pediatric-cancer) (accessed on 2 February 2023).

### 2.2. Patients Sample and Cell Lines

Bone marrow mono nuclear cells (BM-MNCs) from B-ALL and T-ALL patients were recovered by the Biobank of the IRCCS SYNLAB SDN institute [19]. For RT-PCR validations, we used mononuclear cells from healthy cord blood samples. The Jurkat-authenticated human cell line was used. Cell lines were grown in RPMI-1640 supplemented with 10% fetal bovine serum (FBS) and cultured at 37 °C in a humidified atmosphere with 5% CO_2_. The NF-κB reporter (Luc)—HEK293 cell line was purchased from BPS Bioscience (#60650) and grown in RPMI-1640 supplemented with 10% fetal bovine serum (FBS). The ONE-Step™ Luciferase Assay System (BPS-60690-2, Vinci Biochem, Vinci FI, Italy) was used to detect luciferase activity using VictorNivo (Perkin Elmer, Waltham, MA, USA).

### 2.3. RNA Extraction, RT-PCR, and Real-Time PCR

Total RNA from the Jurkat cell line and BM-MNCs of patients were isolated using the Trizol Reagent protocol (Thermo Fischer Scientific, Waltham, MA, USA). cDNA synthesis was performed using the VILO SuperMix kit (Thermo Fisher, Waltham, MA, USA) according to the manufacturer’s instructions. The cDNA concentrations were evaluated using a QubitTM 4 Fluorometer (Thermo Fischer Scientific, Waltham, MA, USA). The RPS18 gene was used for housekeeping.

The oligonucleotides used for RT-PCR were

RPS18fw = 5′-CGATGGGCGGCGGAAAATA-3′;
RPS18rev = 5′-CTGCTTTCCTCAACACCACA-3′;
KCTD1fw= 5′- TGAGTGGTGAGGACACAGTC-3′;
KCTD1rev= 5′- CCTGGTCCCTGCCTAAAGAA-3′;
KCTD3fw= 5′-CATCAGTCCAGCAACTTCCG-3′;
KCTD3rev= 5′-AAGGCCTCATGGAACCGTAA-3′;
KCTD12fw = 5′-TTTCTCCAAGCCCAGCAAAC-3′;
KCTD12rev= 5′-TACAGATAGGCAGCCCTTGG-3′;
KCTD11fw= 5′-GAGGGGAGCCCACATTTTCA-3;
KCTD11rev= 5′-GAGTCTAGTCGGAAGCCGTG-3′;
KCTD15fw = 5′-TGTCATGGCAACAGAACGTG-3′;
KCTD15rev = 5′-CAGAGATCCCACCGCTGTAT-3′.

RT-PCR experiments were performed using a C1000 Touch Thermal Cycler (Bio-Rad, Hercules, CA, USA) using iQ SYBR Green Supermix (1708882, Bio-Rad) and applying the following thermal program: initial denaturation (95 °C, 3 min), 40 cycles of denaturation (95 °C, 10 s), annealing (60 °C, 30 s) and elongation (72 °C, 30 s), final elongation (72 °C, 10 min), and a final hold (4 °C). The melting curve was generated in the range of 60–95 °C. The reaction volume was 25 µL. Each reaction was performed in technical duplicate. Samples were normalized to their RPS18 level using the 2-ΔCt method. Two independent experiments were performed for each RT-PCR. Data were analyzed using Biorad CFX Maestro version 1.0 (Bio-Rad).

### 2.4. Protein Extraction

Cells were dissolved in lysis buffer containing protease inhibitors (Tris HCl pH8 20 mM, NaCl 150 mM, Triton X-100 1%,) and lysed for 30 min at 4 °C. The lysate was centrifuged at 13,000 rpm for 30 min at 4 °C, and the protein content of supernatant was used to determine the protein concentration by colorimetric assay (Biorad, Segrate, Italy).

### 2.5. Western Blotting Analysis

The detailed protocol is provided in [20]. The antibodies used were Anti-KCTD1 (LS-C260993, LS-Bio, Seattle, WA, USA), Anti-Flag (MA1-91878, Thermo Fisher), anti-IKβα (662402, Biolegend, USA), anti-β-catenin (Sc-7963, SantaCruz), and anti-β-actin (ab11004, Abcam, UK) as loading controls. Briefly, 30 μg of protein extracts from each sample were separated by SDS-PAGE and transferred onto a nitrocellulose membrane. The membranes were blocked with 5% non-fat milk in TBS-T (Tris-buffered pH8/0.15% Tween 20) at room temperature for 1 h. Then, they were incubated with primary antibodies diluted in 5% non-fat milk in TBS-T (according to manufacturer instructions) overnight at 4 °C. After three washes in TBS-T, the membranes were incubated with corresponding secondary antibodies for 1 h at room temperature. The signal intensity was visualized using Clarity Max Western ECL Substrate (BioRad cat#1705062) and acquired using the ChemiDoc Imaging system (Bio-Rad, USA). Semi-quantitative densitometric analysis was performed using Image Lab 5.2.1 (Bio-Rad). Protein band normalizations were conducted dividing the signal of the protein of interest to the signal of β-Actin.

### 2.6. KCTD1 Knockdown

KCTD1-sh and negative control plasmids were purchased from the HUSH RNAi catalog (OriGene). Jurkat cells were transfected using the Neon™ Transfection System 100 µL kit (Thermo Fisher Scientific). In detail, 1.5 × 106 cells were harvested, washed once with PBS, and resuspended with 100 µL of Buffer R, added with 10 µg of the plasmid of interest. The mixture was incubated at room temperature for 5′ prior to microporation. To maximize plasmid uptake, the optimization protocol 22 (1400 V, 10 ms, 3 pulses) was used; immediately after electroporation, cells were transferred to a 6-well plate containing 3 mL of pre-warmed RPMI-1640 medium, supplemented with 10% FBS and 2 mM L-glutamine, without the addition of antibiotics. Cell pellets were finally collected after 96 h for further analysis.

### 2.7. Cell Cycle Analyses

Cell cycle analyses were conducted using a DNA-Prep Reagents kit (607055, Beckman Coulter, Brea, CA, USA) with a minimum of 10,000 single-cell events recorded. The percentage of G1, S, and G2/M phases were calculated using the Michael Fox algorithm. Afterward, the analysis of the cell cycle was conducted using Kaluza Analysis Software 2.1 (Beckman Coulter).

### 2.8. Transcriptomic Analyses

RNA-sequencing raw counts were downloaded from Buono et al., 2022 [18]. Normalization and differential expression analysis were performed with DESeq2 [21]. Volcano plot representations were obtained with the R package ggplot2. Unsupervised hierarchical clustering and heatmap representations were performed as in Buono et al. [22]. Expression values were normalized by row to highlight the expression pattern of each KCTD among the four examined conditions. The correlation between KCTD1, KCTD15, and IKβα was calculated through Pearson correlation using gene expression data from the publicly available St. Jude Children’s Research Hospital database.

## 3. Results

### 3.1. KCTD Family Transcriptomic Profile in ALLs

**Figure 1:**
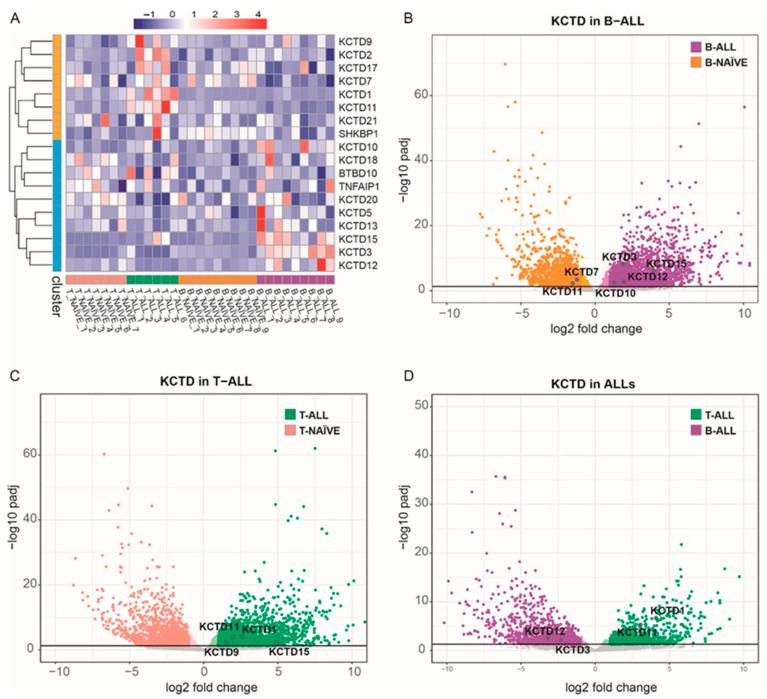
KCTD gene expression in childhood ALL. (**A**) Heat-map shows KCTD gene expression trends in the distinct conditions. Expression values, normalized by row, are indicated with a red (highest expression) to blue (lowest) graded color. Each cluster comprises the KCTDs of a particular condition, one ALL subtype, or the healthy counterparts. Volcano plot illustrating the transcriptome variations between B-ALL and B naïve cells (**B**), T-ALL and T naïve cells (**C**), and B-ALL and T-ALL (**D**). Each dot corresponds to a gene. Light grey dots indicate no significant variations (adjusted *p*-value > 0.05) and significant variations with a log2 (fold change) between 1 and −1. Dark grey dots highlight the position of deregulated KCTDs reported with text in the figure.

Misregulations of the members of the KCTD family in ALL were evaluated by profiling their gene expression through unsupervised hierarchical clustering analysis in 6 T-ALL and 9 B-ALL pediatric patients. Seven T naïve and nine B naïve cell populations from cord blood were used as healthy controls (see methods for details). As shown in Figure 1, the different members of the family presented rather distinct behavior in these pathological contexts, with several KCTDs exhibiting altered expression profiles. The expression profile of seven KCTDs (i.e., KCTD4, KCTD6, KCTD8, KCTD14, KCTD16, KCTD19, and KCNRG) was close to the background in all the samples examined. Therefore, they were considered as not expressed and were not included in the clustering analysis and heatmap representation. Among those dissimilarly expressed in patients and healthy subjects, cluster 1 includes all those KCTDs (KCTD1, KCTD2, KCTD7, KCTD9, KCTD11, KCTD17, and KCTD21) whose expression, although scattered among samples, is increased in T-ALL patients. On the other hand, cluster 2 contains KCTDs that are upregulated in B-ALL patients: KCTD3, KCTD5, KCTD10, KCTD12, KCTD13, KCTD15, KCTD18, and KCTD20. To test the significance of these trends of KCTD expression observed in pediatric acute lymphoblastic leukemia, we performed differential expression analysis among both the ALL subtypes and their relative healthy control. The differential expression analysis between B-ALL samples and the B naïve healthy cells shows that KCTD3, KCTD10, KCTD12, and KCTD15 are significantly upregulated in the B-ALL patients, whereas KCTD7 and KCTD11 present increased expression levels in the healthy control (Figure 1B). On the other hand, the transcriptomic analysis of T-ALL patients highlighted KCTD1, KCTD9, KCTD11, and KCTD15 to be upregulated in pediatric leukemia patients. No KCTDs were found to be downregulated in this pathological state (Figure 1C).

**Figure 2:**
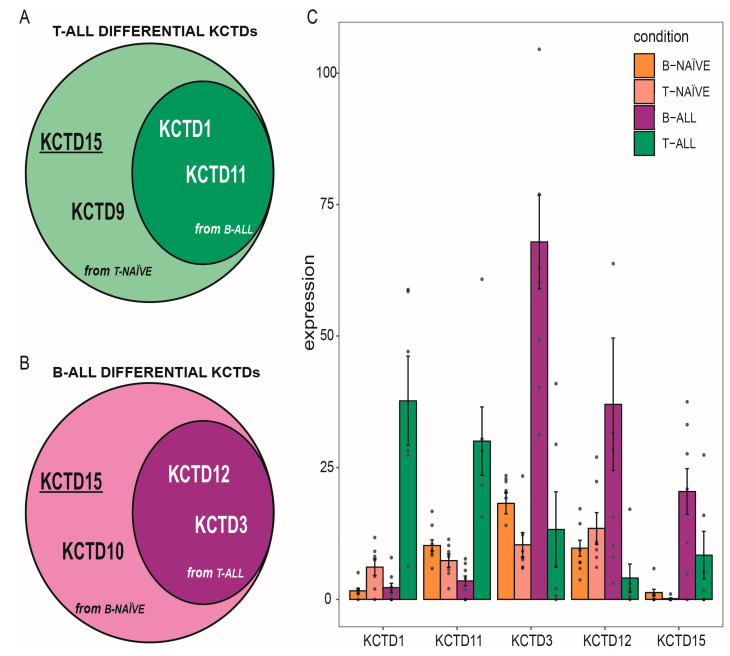
Differentially regulated KCTDs. (**A**) Significantly upregulated KCTDs in T-ALL compared with both B-ALL and healthy subjects. (**B**) Significantly upregulated KCTDs in B-ALL compared with both T-ALL and healthy subjects. (**C**) Gene expression levels of all the significantly differentially expressed KCTDs in all the examined healthy and pathological states. Expression value is expressed as normalized counts from RNA-seq data. Dots represent the expression value for each sample.

Consequently, we compared the expression levels of KCTDs in these two pathological states by directly comparing the transcriptomic landscape of B-ALL and T-ALL childhood patients. These analyses showed that the levels of KCTD3 and KCTD12 are increased in B-ALL while those of KCTD1 and KCTD11 are significantly enhanced in T-ALL patients (Figure 1D). Thus, among all the KCTDs examined, only two pairs of them seem to be able to discriminate not only between the ALL subtype and the relative healthy counterpart, but also between the two types of acute lymphoblastic leukemia: KCTD12 and KCTD3 for B-ALL, and KCTD1 and KCTD11 for T-ALL. Interestingly, KCTD15 increases in both of the hematological types of diseases, suggesting that this protein may have a fundamental role in the carcinogenic transformation of lymphoblasts (Figure 2A,B). It is important to note that KCTD15 is also the protein within this family presenting the highest fold change in comparison with the healthy control in both ALLs (Figure 1B,C). Although other KCTDs may reach high expression values in the pathological condition, they are still moderately expressed in at least one of the healthy samples. Conversely, KCTD15 is the only differentially regulated KCTD that is barely expressed in healthy B naïve lymphocytes from cord blood and not expressed at all in T naïve cells (Figure 2C). The mRNA levels of the KCTDs that are only able to significantly differentiate between B-naïve and B-ALL (KCTD7) and T-naïve and T-ALL (KCTD9 and KCTD10), but not between the two types of ALLs, are shown in Supplementary Figure S1.

### 3.2. KCTDs’ Expression Validation in B-ALL and T-ALL Cohorts

**Figure 3:**
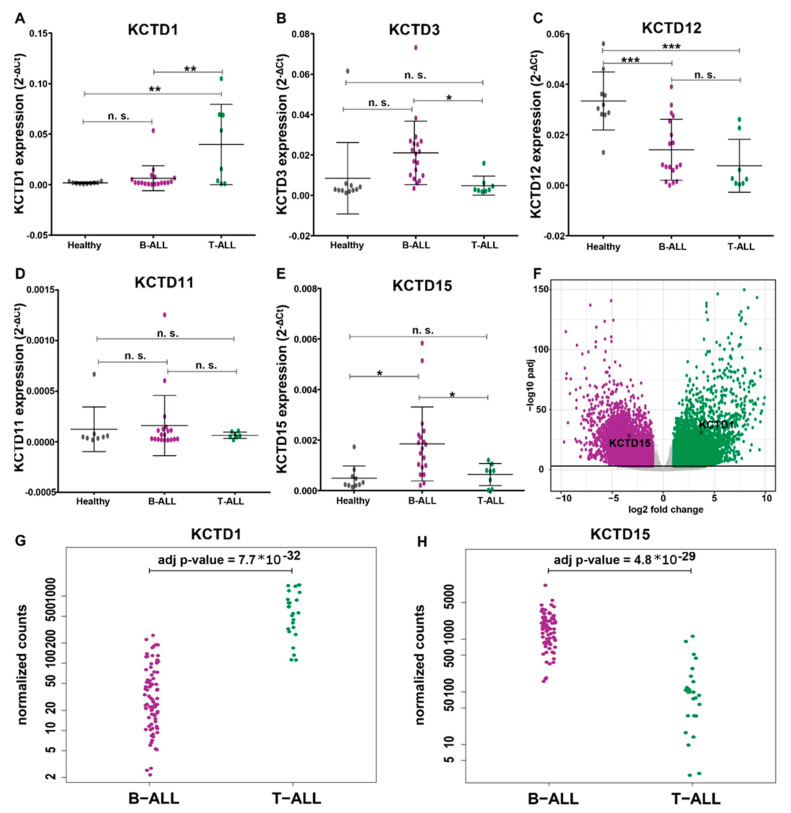
RT-PCR validations on larger cohorts of patients. Expression level of selected KCTD proteins (**A**) = KCTD1, (**B**) = KCTD3, (**C**) = KCTD12, (**D**) = KCTD11, (**E**) = KCTD15 in mononuclear cells from healthy cord blood samples and leukemic cells derived from pediatric B-ALL and T-ALL patients. Expression levels were plotted according to the relative expression (2-ΔCt method) measured in PBMC from healthy donors (*n* = 10, grey circles), in bone marrow cells from diagnosed pediatric B-ALL patients (B-ALL, *n* = 19, purple circles), and in pediatric T-ALL patients (T-ALL, *n* = 8, green circles). * *p* < 0.05, ** *p* < 0.01, *** *p* < 0.001; Mann–Whitney *t*-test. n.s. = not significative. (**F**) Volcano plot illustrating the transcriptome variations between B-ALL and T-ALL using the St. Jude Children’s Research Hospital Database. B-ALL=Purple dots; T-ALL= Green dots. In silico analyses of KCTD1 (**G**) and KCTD15 (**H**) expression levels using data from the St. Jude Children’s Research Hospital database. B-ALL patients: *n* = 99, median age: 15 years. T-ALL patients: *n* = 28, median age: 9 years.

**Figure 4:**
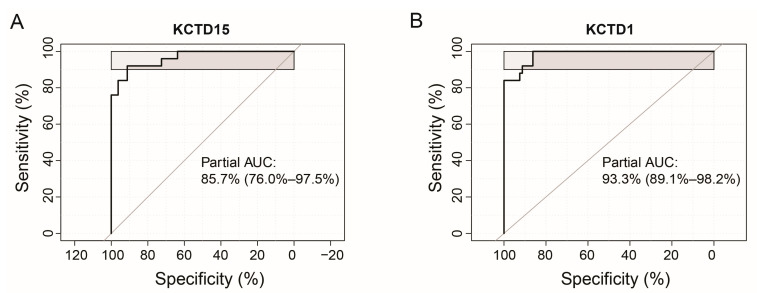
ROC curve of the sensitivity vs. specificity of KCTD15 (**A**) and KCTD 1 (**B**) in B-ALL and T-ALL patients, respectively, reported in the St. Jude Children’s Research Hospital Database.

While naïve B-lymphocytes isolated from cord blood represent an optimal source in studies aimed at highlighting transcriptomic alterations underlying leukemogenesis, these types of samples are not usually associated with diagnostic clinical practices. In order to assess the possibility to exploit the results described in the previous paragraph also in this context, the present investigation was expanded to evaluate the expression levels of five selected KCTDs without isolating any cell types. This study was conducted on a more extended cohort of patients, working on bone marrow samples of 19 pediatric B-ALL patients, 8 pediatric T-ALL patients, and 10 healthy controls, cryo-preserved in our Biobank (see Methods for details). We selected KCTD1, KCTD3, KCTD11, and KCTD12 as the only KCTDs displaying significantly altered values in the transcriptomic analysis comparing B-ALL, T-ALL, and KCTD15, the last being the only one altered in both B-ALL and T-ALL when compared with healthy whole cord blood samples. As seen in Figure 3A, KCTD1 gene expression levels are significantly higher in T-cell leukemias compared with B-cell leukemias and healthy subjects. There are no significant differences between B-ALL and healthy subjects. KCTD3 is able to significantly discriminate between B-cell and T-cell leukemias but not between B-cell leukemias and healthy subjects (Figure 3B). Surprisingly, KCTD12 expression levels are, in these samples, significantly higher in healthy subjects than in both B-ALL and T-ALL subjects, rendering this protein unusable as a potential disease biomarker (Figure 3C). KCTD11 cannot significantly discriminate either between leukemia types or individual leukemia from healthy controls (Figure 3D). Finally, as previously reported [10], the KCTD15 transcript can significantly discriminate against B-ALL subjects from both T-ALL and healthy subjects. However, there were no significant differences between expression levels in T-ALL and control subjects (Figure 3E). These observations are confirmed by an in-silico analysis we performed using data available from the St. Jude Children’s Research Hospital database. As shown in Figure 3F, KCTD15 and KCTD1 are very differentially regulated in B-ALL and T-ALL, respectively. Moreover, the expression level of KCTD1 in T-ALL patients is significantly higher than in B-ALL patients (Figure 3G adj *p*-value = 7.7 × 10^−32^). On the other hand, as previously discussed [10], KCTD15 is significantly more expressed in pediatric B-ALL patients than in T-ALL patients (Figure 3H, adj *p*-value = 4.8 × 10^−29^). In addition, to assess the overall diagnostic performance of KCTD15 in B-ALL and KCTD1 in T-ALL, we performed the ROC curve analyses using the St. Jude Children’s Research Hospital database. As reported in Figure 4A,B, KCTD15 presents a partial area under curve (AUC) of 85.7% while KCTD1 has an AUC of 93.3%, suggesting that both proteins can be considered promising biomarkers capable of discriminating between the two diseases with good sensitivity and specificity.

### 3.3. KCTD1 Knock down Determines a Variation in the Cell Cycle of Jurkat Cells

**Figure 5:**
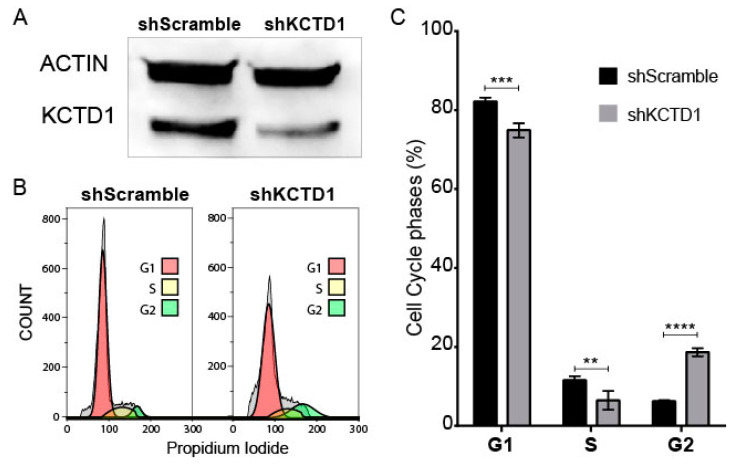
KCTD1 knock down in Jurkat cells. (**A**) Western blot shows the reduction of KCTD1 protein in shKCTD1 treated Jurkat cell line. (**B**) Representative flow cytometry analysis of the cell cycle distribution using Michael H. Fox algorithm. Grey curves are the cells not included in the cell cycle calculation. (**C**) Histogram representation of cell cycle phase percentage in shScramble (black bars) and shKCTD1 (grey bars)-treated Jurkat cells. Error bars report standard deviation of three independent experiments. ** = *p* < 0.01, *** = *p* < 0.001, **** = *p* < 0.0001 Mann–Whitney *t*-test.

We decided to silence KCTD1 in a T-ALL cell line model, the Jurkat cell line, to gain insight into the putative functional role that this protein may have in T-ALL patho-physiology. Using shRNA technology, we were able to effectively reduce KCTD1 levels in Jurkat cells after 96 h from transfection (Figure 5A). The evident reduction of KCTD1 resulted in a change in the cell cycle of the silenced Jurkat cells. Indeed, as observed in Figure 5B,C and Supplementary Figure S2, shKCTD1 Jurkat cells show a significant reduction in G1 and S phases with a corresponding increase in the G2 phase compared with control sh-treated cells. However, the effect of KCTD1 silencing on cell proliferation needs to be experimentally confirmed. These data associate the activity of KCTD1 with an efficient proliferation of cancer T cells, highlighting this protein as a promising therapeutic target.

### 3.4. KCTD1 as Possible Regulator of the NF-κB Pathway

**Figure 6:**
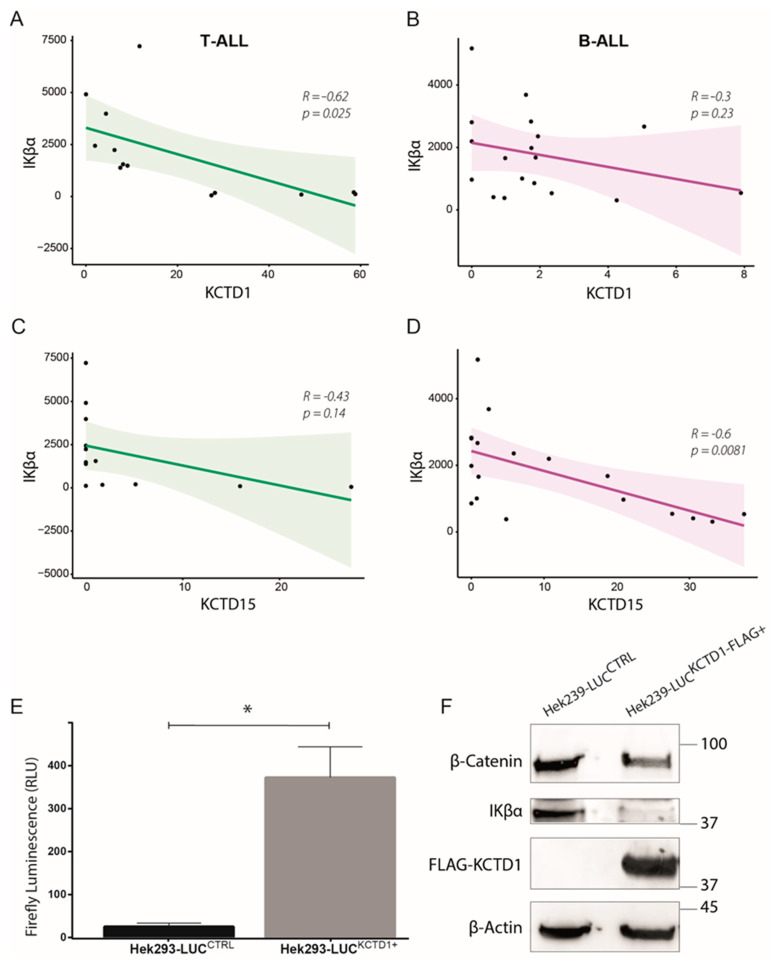
Pearson correlation of gene expression levels expressed in normalized counts between KCTD1 and IKβα in T-ALL (**A**), R = −0.62; *p*-value = 0.025, B-ALL (**B**), R = −0.3; *p*-value = 0.23, patients and between KCTD15 and IKβα in T-ALL (**C**), R = −0.43; *p*-value = 0.14 and B-ALL (**D**), R = −0.6; *p*-value = 0.0081, patients. B. (**E**) Bar-plot diagram of firefly luciferase signal in NF-κB reporter (Luc)-HEK293 control (black bars) and in NF-κB reporter (Luc)-HEK293 over-expressing KCTD1 (grey bars). Errors represent  ±  SD of three independent experiments. * *p*-value  <  0.05, Mann–Whitney *t*-test. β-Catenin, IKβα, KCTD1-FLAG, and β-Actin western blot analyses of NF-κB reporter (Luc)-Hek293 cell line over-expressing KCTD1-FLAG in comparison with control cell line (**F**).

To try to understand the involvement of KCTD1 in T-ALL, we started from recently obtained data on its homolog, the KCTD15 protein [14]. As previously demonstrated, KCTD15 is able to regulate the NF-κB pathway by hyper-activating IKK-β, the kinase responsible for the phosphorylation and subsequent ubiquitination/degradation of IKβα [17]. Given the high sequence similarity between KCTD15 and KCTD1 [16], we questioned whether this similarity could also extend to their pathophysiological role in childhood ALL. Hence, we performed in silico correlation between KCTD1 and IKβα expression using our RNA-seq samples. Our analyses show that while KCTD1 inversely correlates with IKβα significantly in T-ALL (R = −0.62; *p*-value = 0.025) but not in B-ALL (R = −0.43; *p*-value = 0.14), we observed the exact opposite for KCTD15 (Figure 6A–D). This result may suggest a possible involvement of KCTD1 on the NF-κB pathway in T-ALL that could be equivalent to that exerted by KCTD15 in B-ALL. To further elucidate this point, we decided to use the NF-κB reporter (Luc)-Hek293 cell line, a model system for the study of the NF-κB pathway with the promoter for luciferase gene expression under the control of NF- κB. We overexpressed KCTD1-FLAG in this model and, after 16 h, this resulted in a significant and robust increase in the luciferase signal (Figure 6E), unveiling an increase in the NF-κB pathway as KCTD1 increases. Concurrently with the increase in the luciferase signal, the cells exhibited a clear reduction in the levels of IKβα (Figure 6F and Supplementary Figure S3). Further, we also registered a decrease in β-catenin protein expression (Figure 6F and Supplementary Figure S3), an intracellular transducer in the Wnt signaling pathway [23], also known to be suppressed by KCTD1 [24].

## 4. Discussion

KCTDs constitute a heterogenous protein family whose members are involved in many different physio-pathological processes [15,25]. The defining feature of the family is the presence in all of the members of a BTB (broad complex, tramtrak, and bric-a-brac) domain (also denoted as POZ—pox-virus zinc finger—or T1). Although the BTB domains of these proteins present significant sequence similarities with the tetramerization domain of the potassium channel, they are endowed with different structural and functional properties [26,27]. Indeed, not only do they prevalently assume a pentameric state but, rather than favoring the homo-oligomerization process, they are mainly involved in the formation of functional complexes with partners such as Cullin 3, AP-2α, and the GABA-B2 receptor [16,28,29,30]. By exploiting the structural data that emerged from predictive analyses, we have recently unraveled previously undetected similarities among this protein family. These studies have shown that, in most cases, the C-terminal domain also presents significant structural analogies despite the frequent lack of sequence similarities and functional heterogeneity [16]. Based on these findings, a new clustering of the family members has been derived (references and website https://alphafold.ibb.cnr.it/, (accessed on 1 November 2022). In this puzzling scenario, characterized by a difficult definition of structural–functional relationships, here we have performed a comprehensive analysis of all KCTD expression levels in ALL patients. Although several KCTDs do not present altered expression profiles in these pathological states, significant misregulations have been observed for some KCTDs. In addition to the upregulation of KCTD15 in B-ALL we have previously reported [13], the transcriptomic analysis unravels altered profiles for KCTDs belonging to different clusters of the family. Notably, members of the same clusters may or may not have similar misregulation. For example, while KCTD12 and KCTD16 of cluster 1A are upregulated in B-ALL patients, their close homolog KCTD8 is not. Moreover, while KCTD2 and KCTD17 of cluster 3 are upregulated in T-ALL, KCTD5, belonging to the same group, is upregulated in B-ALL. Particularly interesting is the behavior of KCTD1 and KCTD15, two very close proteins (~80% similarity) belonging to cluster 1B. Indeed, while KCTD15 is upregulated in both T-ALL and B-ALL, KCTD1 is specifically upregulated in T-ALL, being barely detectable in both B-ALL patients and healthy subjects (Supplementary Figure S4). These observations have been confirmed by the analysis of cord blood samples of B-ALL, T-ALL, and healthy subjects as well as of model cell lines for these pathologies. In all the examined circumstances, a significant and specific overexpression of KCTD1 in T-ALL was detected. Further, the knock down of this protein in an in vitro model of T-ALL (JURKAT cell line) results in a significant increase in the G2 cell cycle phase. Future experiments using more efficient silencing methods (such as CRISPR/CAS9) will be performed to better understand the involvement of KCTD1 in the regulation of the NF-Kb pathway. Furthermore, the silencing of KCTD1 in the CD3+ cells will be conducted to better understand the role of this protein in physiological conditions as well. A possible molecular mechanism underlying the role of KCTD1 in T-ALL can be inferred from the analysis of literature data. The upregulation of KCTD15 in B-ALL has been associated with the activation of the NF-kB pathway though the activation of the Ikkβ kinase that phosphorylates the NF-kB inhibitor IkBα, marking it for ubiquitination and degradation. Based on high sequence similarities between KCTD1 and KCTD15 and on the detection of a direct binding between recombinant KCTD1 and Ikkβ (Smaldone et al., under review), a similar mechanism can underly the activity of KCTD1 in T-ALL. First, it is important to note that in T-ALLs, the NF- κB pathway is hyperactivated [31]. Using the St. Jude Children’s Research Hospital data, we observed a significant negative correlation between the expression of KCTD1 and IKβα in T-ALLs in contrast to B-ALLs. In contrast, KCTD15 negatively correlates with IKβα expression in B-ALLs but not in T-ALLs. Furthermore, the over-expression of KCTD1 in a model system for studying the NF-κB pathway, the NF-κB reporter (Luc)-Hek293 cell line, resulted in the significant over-activation of this pathway compared with control cells. Our highlights suggest that while in B-ALL, KCTD15 alone sustains the activation of NF- κB; in T-ALL, the two proteins could cooperate to sustain the activity of this pathway during T-ALL pathogenesis events.

## 5. Conclusions

In conclusion, this analysis not only represents the first comprehensive study in which the dysregulation of all KCTDs is simultaneously evaluated in specific pathological contexts, but it also provides a promising target that could serve as both a biomarker and therapeutic target in T-ALL.

## Supplementary Materials

The following supporting information can be downloaded at: https://www.mdpi.com/article/10.3390/jcm12113669/s1, Figure S1: Gene expression levels of KCTD7, KCTD9, and KCTD10 in all the examined healthy and pathological states. The expression value is expressed as normalized counts from RNA-seq data; Figure S2: Gating strategy of the cell cycle of shScramble (top panels) and shKCTD1 (low panels) treated Jurkat cells. On the right, representative flow cytometry analysis of the cell cycle distribution using Micheal H. Fox algorithm; Figure S3: Bar-plot diagram of B-Catenin and IKBα normalized proteins expression (respect to the B-Actin protein band) in Hek293-LUC^ctrl^ (black bars) and Hek293-LUCK^CTD1+^ (grey bars); Figure S4: (A) KCTD1 and β-Actin western blot analyses of Jurkat cells in comparison with CD3+ cells from healthy subjects. Numbers represent the molecular weight of protein marker expressed in kDa. (B) Bar-plot diagram of KCTD1 normalized protein expression in Jurkat cells and CD3+ cells. Error bars represent SD of two independent normalizations.
