# Supplementary material for: A Comprehensive Analysis of the Expression Profiles of KCTD Proteins in Acute Lymphoblastic Leukemia: Evidence of Selective Expression of KCTD1 in T-ALL
Source: J Clin Med. 2023 May 25;12(11):3669. doi: 10.3390/jcm12113669 (PMC10253327; doi:10.3390/jcm12113669)
Supplement: Supplementary file 1 [file jcm-12-03669-s001.zip › jcm-2347927-supplementary.pdf]

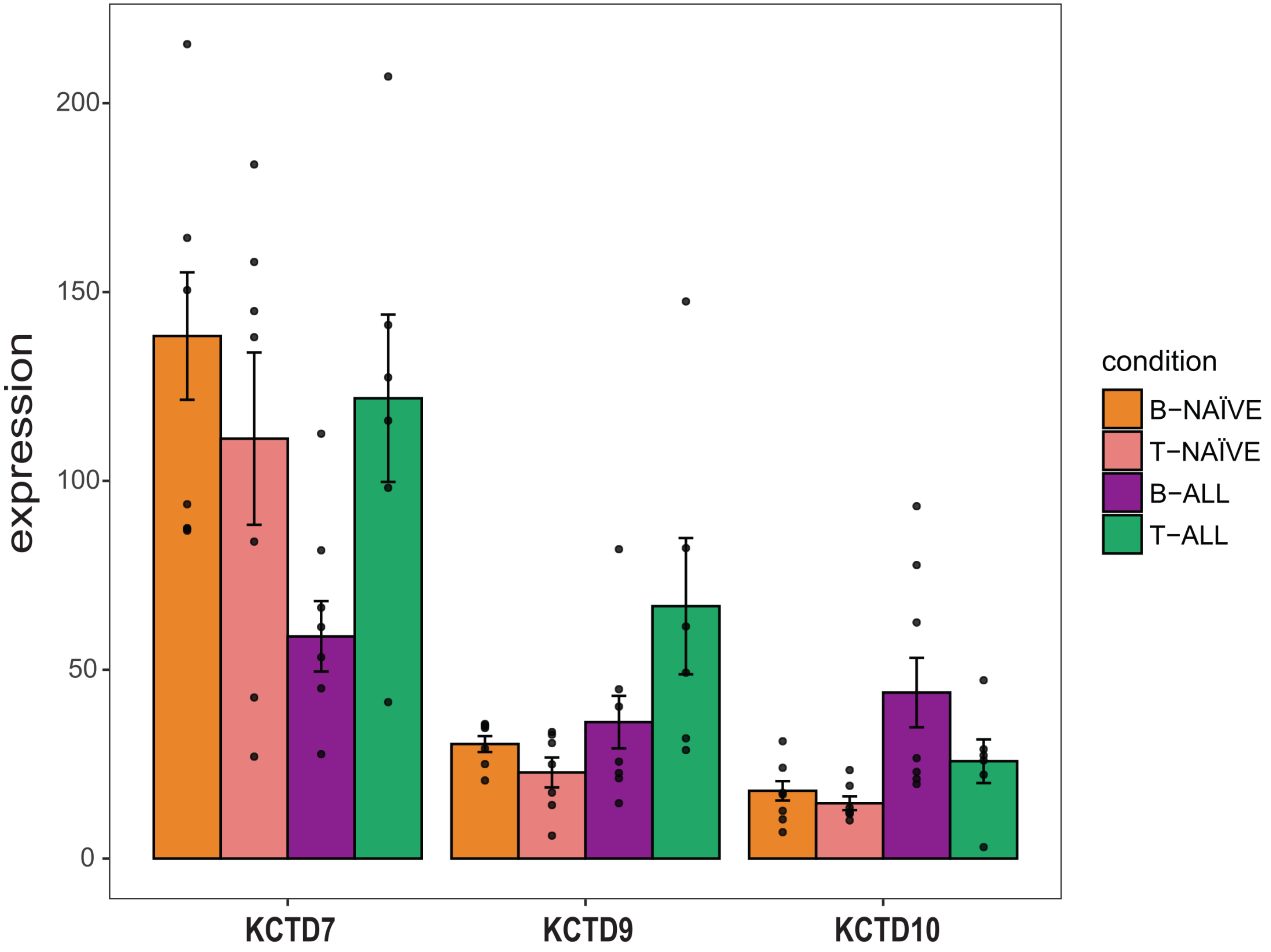

Supplementary Figure S1. Gene expression levels of KCTD7, KCTD9 and KCTD10 in all the examined healthy and pathological state. Expression value is expressed as normalized counts from RNA-seq data.

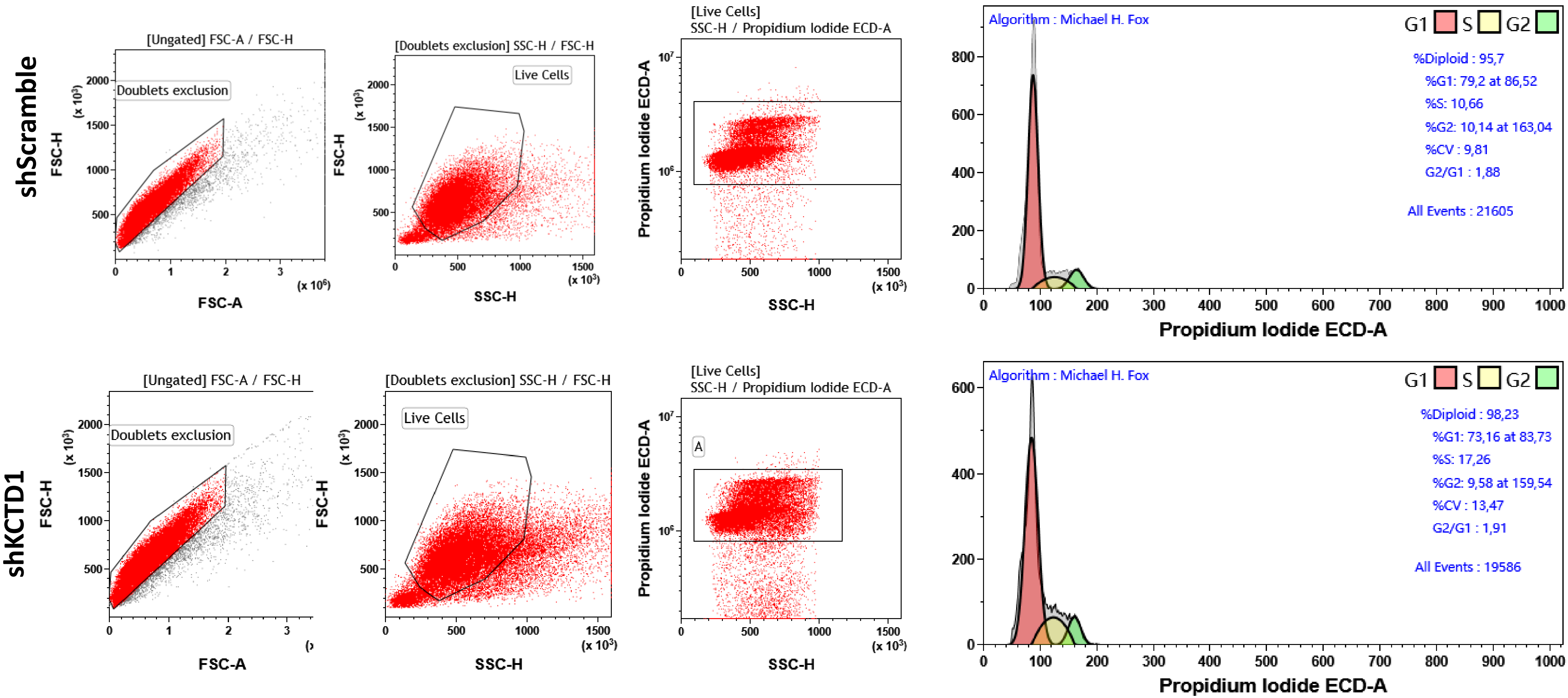

Supplementary Figure S2. Gating strategy of cell cycle of shScramble (top panels) and shKCTD1 (low panels) treated Jurkat cells. On the righth, representative flow cytometry analysis of the cell cycle distribution using Micheal H. Fox algoritm.

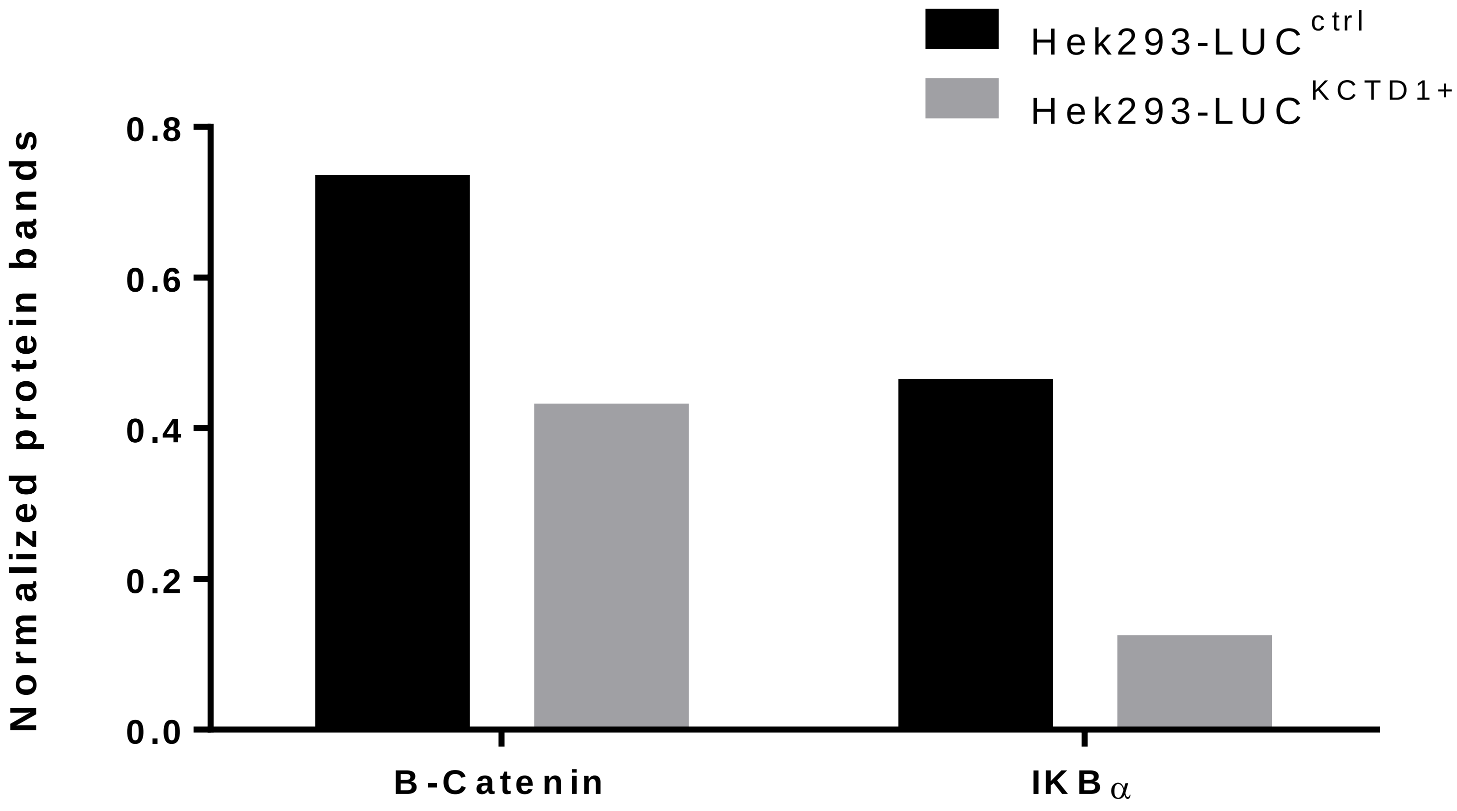

Supplementary Figure S3. Bar-plot diagram of B-Catenin and IKBα normalized proteins expression (respect to the B-Actin protein band) in Hek293-LUC^{ctrl} (black bars) and Hek293-LUC^{KCTD1+} (grey bars).

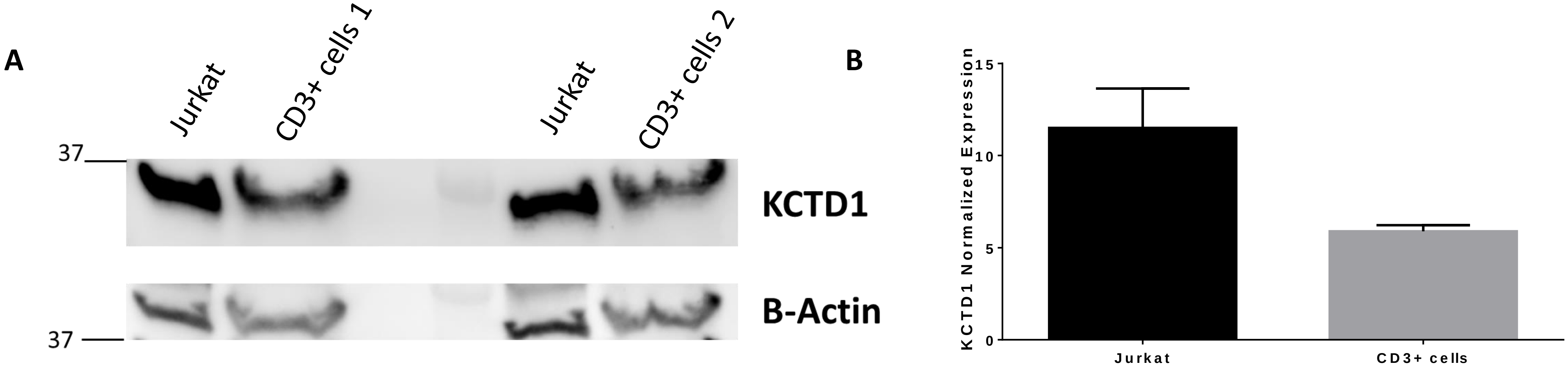

Supplementary Figure S4.A) KCTD1 and β-Actin western blot analyses of Jurkat cells in comparison with CD3+ cells from healthy subjects. Numbers represent molecular weight of protein marker expressed in kDa. B) Bar-plot diagram of KCTD1 normalized protein expression in Jurkat cells and CD3+ cells. Error bars represent SD of two independent normalizations.
